# Knockout of *VvCCD8* gene in grapevine affects shoot branching

**DOI:** 10.1186/s12870-020-2263-3

**Published:** 2020-01-29

**Authors:** Chong Ren, Yuchen Guo, Junhua Kong, Fatma Lecourieux, Zhanwu Dai, Shaohua Li, Zhenchang Liang

**Affiliations:** 1 Beijing Key Laboratory of Grape Science and Enology, the Chinese Academy of Science, Beijing, 100093 People’s Republic of China; 2 CAS Key Laboratory of Plant Resources, Institute of Botany, the Innovative Academy of Seed Design, the Chinese Academy of Science, Beijing, 100093 People’s Republic of China; 3 University of Chinese Academy of Sciences, Beijing, 100049 People’s Republic of China; 4 EGFV, Bordeaux Sciences Agro, INRA, Université de Bordeaux, ISVV, 33140 Villenave d’Ornon, Bordeaux, France; 5 Institute of Botany, the Chinese Academy of Sciences, Nanxin Village 20, Xiangshan, Haidian District, Beijing, 100093 China

**Keywords:** CRISPR/Cas9, CCD8, Mutant, Shoot branching, Strigolactone

## Abstract

**Background:**

Shoot branching is an important trait of plants that allows them to adapt to environment changes. Strigolactones (SLs) are newly identified plant hormones that inhibit shoot branching in plants. The SL biosynthesis genes *CCD7* (carotenoid cleavage dioxygenase 7) and *CCD8* have been found to regulate branching in several herbaceous plants by taking advantage of their loss-of-function mutants. However, the role for *CCD7* and *CCD8* in shoot branching control in grapevine is still unknown due to the lack of corresponding mutants.

**Results:**

Here we employed the CRISPR/Cas9 system to edit the *VvCCD7* and *VvCCD8* genes in the grape hybrid 41B. The 41B embryogenic cells can easily be transformed and used for regeneration of the corresponding transformed plants. Sequencing analysis revealed that gene editing has been used successfully to target both *VvCCD* genes in 41B embryogenic cells. After regeneration, six 41B plantlets were identified as transgenic plants carrying the *CCD8*-sgRNA expression cassette. Among these, four plants showed mutation in the target region and were selected as *ccd8* mutants. These *ccd8* mutants showed increased shoot branching compared to the corresponding wild-type plants. In addition, no off-target mutation was detected in the tested mutants at predicted off-target sites.

**Conclusions:**

Our results underline the key role of *VvCCD*8 in the control of grapevine shoot branching.

## Background

The control of shoot branching is an adaptive strategy that allows plants to optimize their growth to adapt to environmental changes. Shoot branching is determined by the number and outgrowth of axillary buds, and bud outgrowth contributes to the flexibility in branching [1]. Auxin and cytokinin are master regulators that control shoot branching in plants. Auxin was considered as inhibitor in bud outgrowth [2, 3], whereas cytokinin was found to promote this process [2]. However, the established hormone signaling pathways cannot fully explain the control of bud outgrowth [4], suggesting the existence of other regulators.

Strigolactones or their derivates (SLs) are newly identified plant hormones that suppress axillary bud outgrowth [5, 6]. SLs are a group of molecules synthesized from carotenoids. Two carotenoid cleavage dioxygenases, CCD7 and CCD8, have been shown to be required for SLs biosynthesis [6, 7]. CCD7 and CCD8 are also known as MORE AXILLARY BRANCHING3 (MAX3) and MAX4 in *Arabidopsis* [7, 8]. CCD7 and CCD8 orthologs have also been identified in the strigolactone biosynthetic pathway of several plant species, such as DWARF17 (D17) and D10 in rice [9–11], RAMOSUS5 (RMS5) and RMS1 in pea [8, 12] and DECREASED APICAL DOMINANCE3 (DAD3) and DAD1 in petunia [13, 14]. These orthologous proteins were found to be involved in branching control, and a highly branched phenotype has been reported in the corresponding loss-of-function mutants [15, 16]. Additionally, mutations in the α/β-fold hydrolase D14 that functions as a SL receptor in *Arabidopsis* and rice resulted in an increased shoot branching phenotype [17–19]. SLs inhibited bud outgrowth by increasing the expression of *BRANCHED1* (*BRC1*), which encodes a bud outgrowth repressor [20–22]. Loss-of-function mutations in *BRC1* affected bud outgrowth and resulted in increased shoot branching [20, 23]. Likewise, in poplar, knockdown of *BRC1* affected shoot architecture [24].

Recently, SLs were proposed to control scion development in response to nitrogen availability in grafted grapevine plants [25]. Additionally, overexpression of grape *CCD7* or *CCD8* gene in *Arabidopsis max3* or *max4* mutants background partly reverted their phenotypes [25], suggesting a potential role for *CCD7* and *CCD8* in grapevine shoot branching. However, up to date, in grapevine no experimental evidence supporting the role of these two genes in the control of shoot branching exists. This role has therefore still to be demonstrated in grapevine. CRISPR/Cas9 (clustered regulatory interspaced short palindromic repeats/CRISPR-associated protein 9) system is a powerful tool for targeted mutagenesis that has been successfully applied in many plant species to achieve genome editing. In grape, this system was efficiently used to edit *IdnDH* (L-idonate dehydrogenase), *PDS* (phytoene desaturase), and *VvWRKY52* genes [26–28]. This indicates that the CRISPR/Cas9 system can be used for precise genome editing in grapevine.

In this study, we used the CRISPR/Cas9 technology to edit the *VvCCD7* and *VvCCD8* genes in 41B grapevine rootstock, respectively. As 41B embryogenic cell transformation, selection and regeneration are easy to perform, these cells were chosen to perform gene editing experiments. After regeneration, four *VvCCD8* knockout lines were obtained. The recovered *ccd8* mutants exhibited increased shoot branching when compared to wild-type plants. Sanger sequencing results showed that *VvCCD8* mutant plants carried the targeted mutations, and that no mutation occurred at the putative off-target sites. Altogether, these results underline the efficiency of grape genome editing and provide evidence that *VvCCD8* plays a key role in the control of shoot branching in grapevine.

## Results

### Target design and CRISPR/Cas9 vector construction

**Fig. 1:**
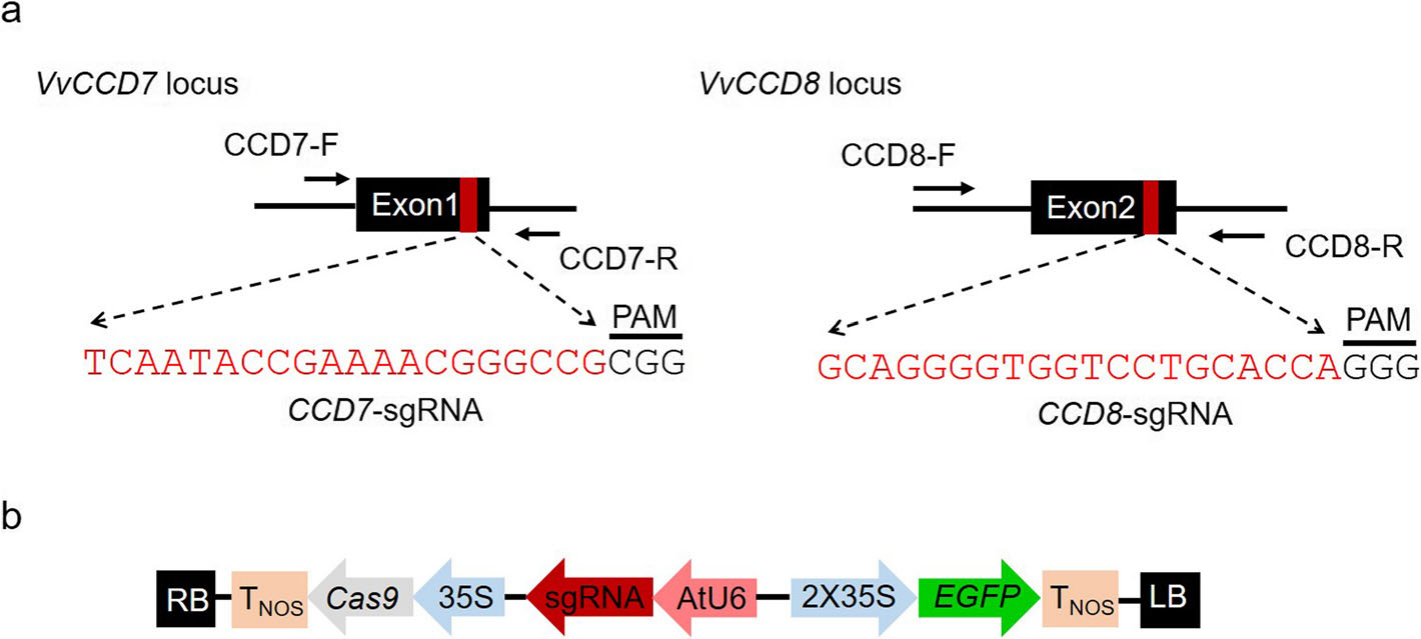
Schematic illustration of target design and the binary vector. **a** Schematic map of the target sites within *VvCCD7* and *VvCCD8* genes. The sequences of sgRNAs are indicated in red. CCD7-F/R and CCD8-F/R are primers used for PCR amplification. **b** Schematic diagram of the revised pCACRISPR/Cas9 vector. The *EGFP* reporter gene was used for rapid selection of transformed cells after transformation. 35S, CaMV35S promoter; AtU6, Arabidopsis small RNA U6 promoter; T_NOS_, nopaline synthase terminator; RB, right border; LB, left border

The *VvCCD7* (VIT_15s0021g02190) and *VvCCD8* (VIT_04s0008g03380) genes contain 6 and 5 exons, respectively. Considering that targeted mutagenesis caused by CRISPR/Cas9 generally resulted in frameshifts or generation of stop codons [26, 27], the upstream exons would be better targets for gene editing to produce non-functional proteins. Thus, the first exon (Exon1) of *VvCCD7* and the second exon (Exon2) of *VvCCD8* were selected as the targets for CRISPR-Cas9 gene editing, respectively (Fig. 1a). The target regions of these two genes were cloned and verified by Sanger sequencing prior to sgRNA design. The results showed that the amplified sequences of *VvCCD7* and *VvCCD8* are almost identical to their reference sequences (Additional file 1: Figure S1). The sgRNAs used for targeting *VvCCD7* (*CCD7*-sgRNA) and *VvCCD8* (*CCD8*-sgRNA) were designed accordingly (Fig. 1a). Both sgRNAs were driven by the *Arabidopsis* U6 promoter (AtU6), while the expression of *Streptococcus pyogenes Cas9* was under the control of CaMV35S promoter (35S). The *EGFP* (enhanced green fluorescent protein) gene was used as a reporter gene to rapidly select efficiently transformed cells (Fig. 1b).

### Targeted mutagenesis of *VvCCD7* and *VvCCD8* genes in 41B cells

**Fig. 2:**
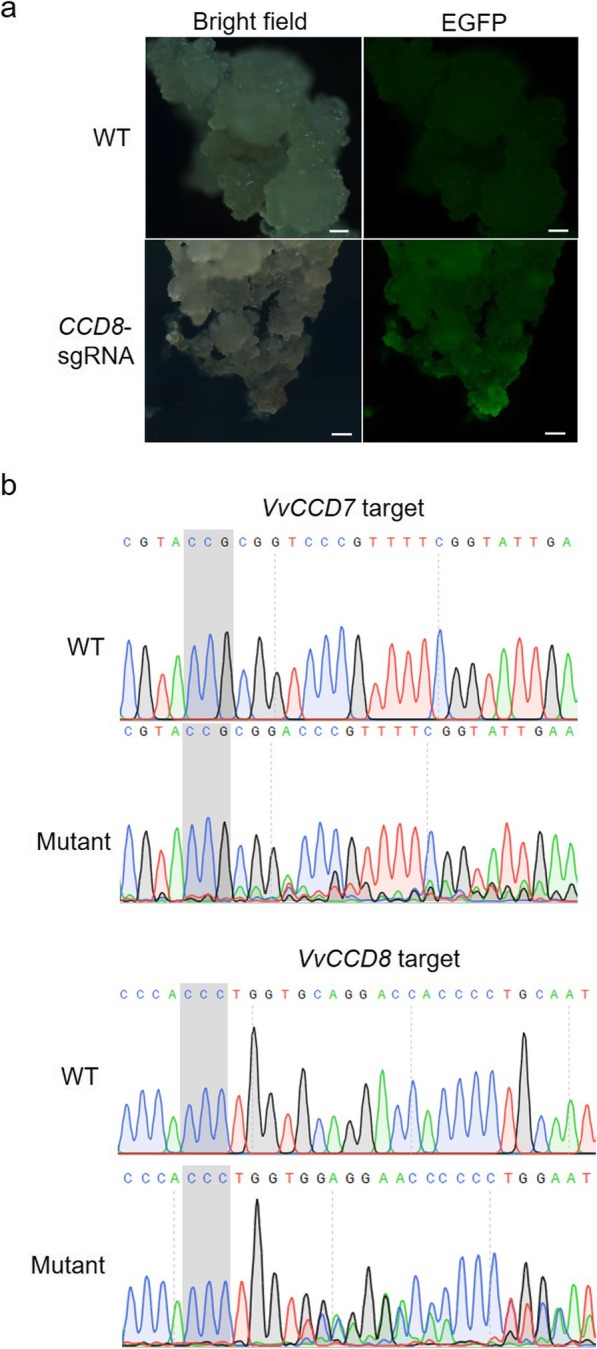
Detection of mutagenesis in transformed 41B cells. **a** Detection of EGFP signal in 41B cells. The cells transformed with *CCD8*-sgRNA expression construct was given as an example. Those cells with EGFP signal were considered as transformed cells and were used for subsequent analysis. Scale bars: 100 μm. **b** Sanger sequencing results of the target sites in *VvCCD7* and *VvCCD8* genes in transformed 41B cells. The wild-type sequences generated clear sequencing chromatograms, whereas the mutated sequences generated overlapping peaks at the mutation sites. The PAM sequences adjacent to *CCD7*-sgRNA and *CCD8*-sgRNA are shadowed

The constructed CRISPR/Cas9 expression vectors were introduced into 41B grape cells by *Agrobacterium*-mediated transformation. Successfully transformed cells were selected by EGFP fluorescence, whereas no fluorescence signal could be detected in untransformed cells (Fig. 2a). The 41B cells exhibiting EGFP signal were sampled and subjected to Sanger sequencing in order to reveal the presence of mutations at the target sites. The sequencing chromatograms were manually analyzed for the presence of double tracing peaks at the target regions, considering that the presence of overlapping peaks was a typical indicator of targeted mutations [29]. Our sequencing results (Fig. 2b) revealed the presence of overlapping peaks in the positively transformed 41B cells by contrast to the single peaks of wild-type (WT) cells chromatograms. These results clearly indicate the existence of targeted mutagenesis in *VvCCD7* and *VvCCD8* genes in transformed 41B cells.

### *VvCCD8* knockout lines show increased shoot branching phenotype

**Fig. 3:**
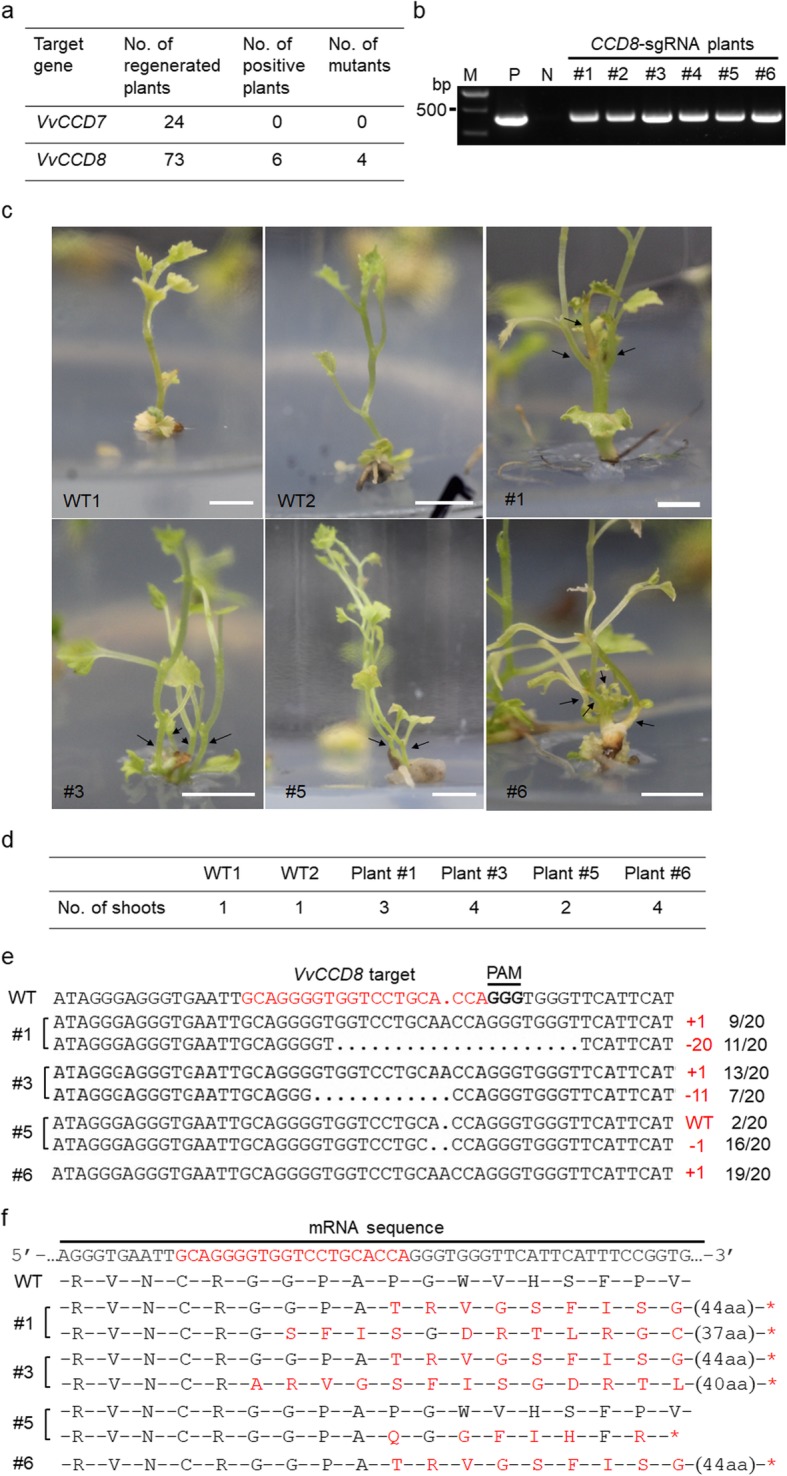
Identification of *VvCCD8* knockout mutants. **a** Overview of identification of regenerated plants. **b** Identification of exogenous T-DNA insertions in regenerated plants by PCR. The specific primers designed for *Cas9* gene were used for PCR identification. Only *CCD8*-sgRNA plants were identified with exogenous T-DNA insertions. Lanes 1–6 represent different individual *CCD8*-sgRNA plants. The plasmid was used as the positive control (P), while the wild-type genomic DNA was used as the negative control (N). M, DNA marker. The cropped gel image is shown here, and the original, uncropped image is available in Additional file 3: Figure S3. **c** Phenotypes of *VvCCD8* knockout mutants. The shoot branches of *VvCCD8* knockout mutants were indicated in black arrows. Scale bars: 0.5 cm. **d** The branch number of the four *VvCCD8* knockout mutants. e Sequencing results of the target sites in the four *VvCCD8* knockout mutants. The gene fragments were amplified from each mutant plant and were cloned into pLB vector for Sanger sequencing assay. A number of 20 clonal amplicons for each plant were analyzed. The mutated sequences identified from the mutants were shown. The plant IDs are shown on the left. Mutation types (colored in red) and the corresponding number (indicated in black) of clones were shown on the right. Those undesired sequences were omitted from the analysis. **f** Mutations of amino acids in mutated sequences shown in **e**. The altered amino acids are colored in red and the premature stop codons are indicated in red asterisks (*). The number of amino acids (aa) that are not shown in the figure is indicated in parentheses

The EGFP-fluorescent 41B cells were used for plant regeneration. A number of 24 and 73 regenerated plants were obtained for *CCD7*-sgRNA and *CCD8*-sgRNA, respectively (Fig. 3a). The recovered plants were selected by PCR using *Cas9*-specific primers (Additional file 4: Table S1). The PCR results showed that among *CCD8*-sgRNA regenerated plants 6 plants contained the exogenous *Cas9* gene (Fig. 3b), indicating a transformation rate of 8.2% (6/73). By contrast, none of the 24 *CCD7*-sgRNA selected plants presented the exogenous *Cas9* gene (Fig. 3a). Among the 6 *CCD8*-sgRNA plants, four (Plant #1, Plant #3, Plant #5 and Plant #6) were identified as *ccd8* mutants (Fig. 3a). Interestingly, all *ccd8* mutants showed increased shoot branching, with Plant #3 and Plant #6 containing 4 shoots, Plant #1 containing 3 shoots, and Plant #5 containing 2 shoots (Fig. 3c and d). In these mutant plants, *VvCCD8* target sequences were analyzed by Sanger sequencing. Twenty clones of PCR amplicons were sequenced for each mutant plant. The results showed that Plant #1 and Plant #3 contained two types of mutations at the target site. The first one corresponds to an insertion of one nucleotide and the second one to a deletion of several nucleotides (20 bp for Plant #1 and 11 bp for Plant #3) (Fig. 3e). These results suggest that these two mutant plants might be biallelic. According to the sequencing results, Plant #5 and Plant #6 only contained one type of mutation (Fig. 3e). Plant #6 mutant might be homozygous as almost all its sequenced clones (19/20) contained the same mutation (1-bp insertion). By contrast, Plant #5 mutant might be heterozygote or chimeric as both wild-type and mutated (1-bp deletion) sequences were identified within the sequenced clones (Fig. 3e). These different mutations led to frameshifts changes, resulting in new mutated amino acid sequences (Fig. 3f) or in the production of stop codons (Fig. 3f) that would result in premature termination of translation.

**Fig. 4:**
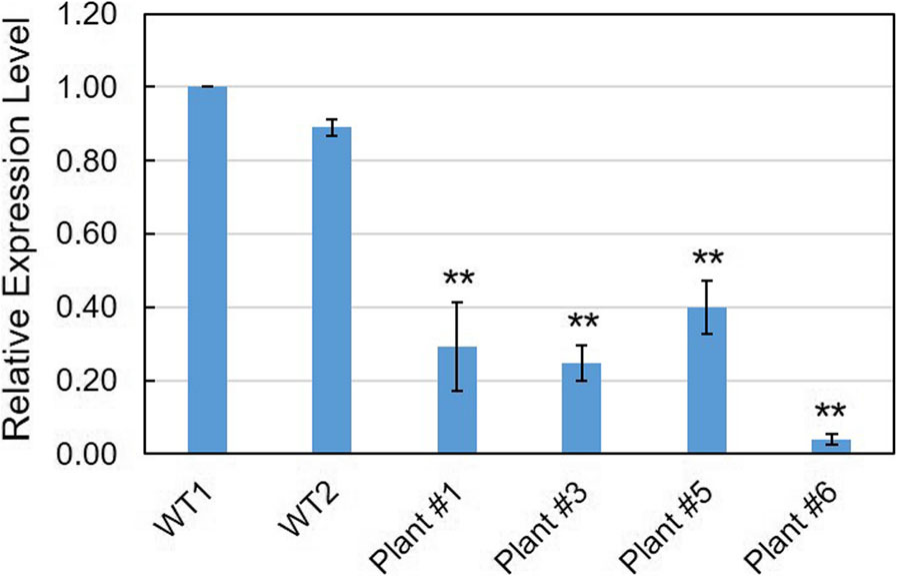
Expression profiles of *VvCCD8* in *ccd8* mutant plants. The expression of *VvCCD8* was determined by quantitative real-time PCR, and the *Actin 1* (accession no. AY680701) was used as internal control. The experiment was repeated three times and the data are shown as mean ± SD. The significance of differential expression level was examined by Student’s *t*-test with *P* < 0.01 indicating highly significant (**)

We also investigated the expression profiles of *VvCCD8* in these mutant plants. The results showed that the transcript abundance of *VvCCD8* in the four mutants was significantly decreased in comparison with wild-type plants (Fig. 4), suggesting that the targeted mutagenesis observed in *VvCCD8* resulted in transcript decay in these *ccd8* mutants.

### Off-target effect was not detected in *ccd8* mutants

To make sure that the enhanced shoot branching phenotype observed in *ccd8* mutants was not due to off-target effects, we investigated the putative genomic off-target loci of *CCD8*-sgRNA in Plant #1 and Plant #6. Putative off-target sites were predicted according to their sequence homology with *CCD8*-sgRNA. Among the 5 highest ranked potential off-target sites, 2 sites were predicted to localize in the exons of VIT_03s0091g00830 and VIT_13s0019g01150 sequences (Additional file 5: Table S2). These two putative off-target sites were therefore selected for further analysis. DNA fragments containing the 2 putative off-target sites were amplified from Plant #1 and Plant #6 by PCR using specific primers (Additional file 4: Table S1). PCR products were cloned into pLB vector and verified by Sanger sequencing. No mutation was detected at the potential off-target sites (Additional file 2: Figure S2), supporting the fact that *VvCCD8* editing was efficient in grapevine and that the increased branching phenotype observed in *ccd8* mutants was specifically due to the mutation in *VvCCD8* and not to off-target effects.

## Discussion

The CRISPR/Cas9 system has emerged as a powerful tool for genome editing, and it shows great potential in generating mutants in plants. In *Arabidopsis,* CRISPR/Cas9 system was successfully used to produce *cbfs* mutants. The characterization of these mutants revealed the important role played by CBF2 in cold acclimation-dependent freezing [30]. Intriguingly, targeted mutagenesis of SBP-CNR and NAC-NOR transcription factors, which are thought to be master regulators of tomato ripening, resulted in tomato delayed ripening or partial non-ripening. This phenotype was surprisingly distinct from the previously used original tomato mutants [31], suggesting a great potential of CRISPR/Cas9 system in gene functional research. SLs were found to retard bud outgrowth [5, 6], and SL biosynthetic genes *CCD7* (*MAX3*) and *CCD8* (*MAX4*) had been demonstrated to be involved in branching control in multiple herbaceous plants, including *Arabidopsis*, rice and tomato [8, 32–34]. In woody plant, poplar *MAX4* (*CCD8*) knockdown lines exhibited altered branching patterns [24]. In grape, it has been suggested that SL could be involved in the control of scion architecture in grafted grapevine plants, based on the observation that exudate from grape *CCDs*-overexpressing transgenic cells could stimulate the germination of *Phelipanche ramosa* seeds, and that overexpression of grape *CCD7* or *CCD8* gene in corresponding *Arabidopsis* mutant can partly revert the mutant phenotype [25]. However, no direct and clear evidence supporting this role exists in grapevine.

In the present study, we employed the CRISPR/Cas9 system to edit *VvCCD7* and *VvCCD8* genes in grapevine (41B rootstock). After transformation of 41B embryogenic cells, Sanger sequencing assay was performed to detect the targeted mutations. The results showed that the designed sgRNAs could effectively direct the targeted editing in both genes (Fig. 2b). The whole plants were obtained through regeneration, and transgenic plants were identified and selected by PCR. Only 6 *CCD8*-sgRNA plants were found to contain exogenous *Cas9* gene (Fig. 3b). The lack of antibiotics-dependent selection and low regeneration rate of 41B cells in this experiment are probably responsible for low rate of transgenic plants obtained. Among the 6 transgenic plants, 4 were identified as *ccd8* mutants (Fig. 3a). As expected, the *ccd8* mutants exhibited increased shoot branching, which is in agreement with previous reports [13, 15, 16, 24]. Except for Plant #5, all mutants had at least 3 shoots, whereas WT plants generally had only one shoot (Fig. 3d). According to the sequencing results, Plant #5 might be heterozygous or chimeric (Fig. 3e), suggesting a possible relationship between the number of shoots and SLs concentration in grapevine. Off-target effect is a major concern when applying CRISPR/Cas9 technology. We therefore investigated the putative genomic off-target loci of *CCD8*-sgRNA, and no off-target mutation was observed (Additional file 2: Figure S2). These results ruled out the possibility that the altered shoot branching observed in *ccd8* mutants was caused by the presence of off-target mutations.

Interestingly, the *ccd8* mutants obtain with 41B rootstock could serve for grafting experiments in order to further study the role of SLs in the control of grapevine shoot branching. Finally, whether *VvCCD7* plays a same role in shoot branching is remains unclear and has yet to be investigated. To go further in this direction, new attempt to regenerate *ccd7* mutant plants could be achieved in the future.

## Conclusions

Collectively, our results showed that CRISPR/Cas9 system can be successfully used to knock out *VvCCD7* and *VvCCD8* genes in grape. Additionally, the study of *VvCCD8* knockout grapevine plants revealed the key role of this gene in the control of shoot branching, therefore providing a first clue to investigate the mechanisms involved in the regulation of shoot architecture in grapevine.

## Methods

### Design of sgRNA and construction of genome editing vectors

The target regions of *VvCCD7* and *VvCCD8* genes were amplified from 41B embryogenic cells by PCR with primers CCD7-F/R and CCD8-F/R, respectively. The amplified fragments were verified by Sanger sequencing. The verified sequences were used as an input for sgRNA design with the online tool CRISPR-P v2.0 [35]. The potential off-target sites were predicted simultaneously with this tool. The designed sgRNAs were then ligated into the pCACRISPR/Cas9 vector via homologous recombination (HR). PCR cloning, sgRNA design and plasmid construction were conducted as previously described [26]. The pCACRISPR/Cas9 vector was digested with *Sma*I and *Xho*I to remove the *hpt*II (hygromycin phosphotransferase II) gene, and the *EGFP* gene (NCBI accession: NC_025025) amplified from pCAMBIA2300-EGFP vector was inserted into the linearized pCACRISPR/Cas9 vector via HR using the ClonExpress II One Step Cloning Kit (Vazyme, China). The primers used in the experiment are available in Additional file 4: Table S1.

### Plant material, transformation and regeneration

The embryogenic grape cells derived from 41B rootstock (*Vitis vinifera* cv. Chasselas × *Vitis berlandieri*) were graciously provided by Dr. F. Lecourieux (EGFV, Université de Bordeaux), and the cells were cultured as previously described [36]. In brief, the suspension cells were subcultured weekly in 25 mL of liquid glycerol-maltose (GM) medium containing 1 mg L^− 1^ naphthoxy acetic acid (NOA) in the dark.

The constructed binary vectors were introduced into *Agrobacterium tumefaciens* strain EHA105 by the freeze-thaw method, and the 41B embryogenic cells were transformed using the *A. tumefaciens* co-cultivation method [37]. After co-cultivation, the grape cells were first washed twice with liquid GM medium and then subcultured every other day in GM medium supplemented with 200 mg/L timentin for 1 week. Then the cells were collected and divided into small groups (~ 0.5 cm^2^) for EGFP detection.

For induction of embryogenesis, 41B cells were transferred onto solid hormone-free regeneration medium (GM medium without NOA) under a 16-h photoperiod with white fluorescent lights. Plants regenerated on McCown woody plant medium (Duchefa) supplemented with 3% sucrose, 0.2 mg/L naphthalene acetic acid (NAA), 0.5 mg/L activated charcoal, 7.5 g/L agar under long-day (16 h light/8 h dark) conditions.

### Extraction of genomic DNA and PCR identification of exogenous T-DNA insertion

Genomic DNA was prepared using the CTAB plant genomic DNA extraction kit (Aidlab, China) according to the manufacturer’s instructions. The isolated DNA was used as the template for PCR. The PCR reaction was performed with *Cas9*-specific primers (Additional file 4: Table S1) using Es Taq DNA polymerase (CWBIO, China) according to the manufacturer’s protocol. The PCR products were detected by 1% agarose gel electrophoresis and were further confirmed by Sanger sequencing.

### Sanger sequencing assay

The DNA fragments containing the target sites were amplified from 41B cells or regenerated plants by PCR with the primers CCD7-F/R and CCD8-F/R, respectively. The PCR products amplified from grape cells were purified and directly used for Sanger sequencing analysis (Tsingke, Beijing). The amplified fragments from 41B plants were cloned into pLB-Simple vector (TIANGEN, China), and a total of 20 clones for each sample were sequenced.

### Quantitative real-time PCR assay

The expression profiles of *VvCCD8* gene were investigated using quantitative real-time PCR (qRT-PCR) with *VvCCD8* specific primers (Additional file 4: Table S1). The grape *Actin 1* (accession no. AY680701) was used as internal control, and the relative expression level was determined using the 2^−ΔΔCT^ method [38]. The qRT-PCR assay was performed as previously reported [26].

### Off-target analysis

Off-target analysis was performed in *VvCCD8* knockout lines. Two top-ranking putative off-target sites that localize in gene exons were chosen for off-target analysis. The potential off-target regions were amplified using their specific primers (Additional file 4: Table S1), and the fragments were cloned into pLB vector and at least 6 clones were analyzed by Sanger sequencing.

### EGFP detection

The EGFP signal was detected using the Eclipse Ni-U fluorescence microscope (Nikon, Japan) with excitation at 487 nm, emission at 505 nm. The wild-type cells were used as the negative control.

## Supplementary information

**Additional file 1: Figure S1.** Sequencing results of *VvCCD7* and *VvCCD8* fragments amplified from 41B cells. **a** The target sequence of *VvCCD7* gene in 41B. **b** The target sequence of *VvCCD8* gene in 41B. The target sites in *VvCCD7* and *VvCCD8* genes are highlighted in dark blue.

**Additional file 2: Figure S2.** Sequencing results of the two putative off-target sites in *VvCCD8* knockout lines. The two off-target sites predicted within exons of other genes were selected for off-target analysis. Two *VvCCD8* knockout lines, Plant #1 and Plant #6 were used in the experiment. The amplified fragments containing the off-target sites were amplified and cloned into pLB-Simple vector. At least 6 clones for each site were used for Sanger sequencing.

**Additional file 3: Figure S3.** The original gel image of PCR identification of T-DNA insertions in *CCD8*-sgRNA plants. The vector plasmid (P1) and the transgenic cells (P2) were used as the positive controls, while wild-type plant was used as the negative control (N). Lanes 1–6 represent individual *CCD8*-sgRNA plants.

**Additional file 4: Table S1.** List of primers used in this study.

**Additional file 5: Table S2.** Putative off-target sites predicted for *CCD8*-sgRNA.

## Data Availability

The datasets supporting the conclusions of this article and materials used in this study are available by contacting with the corresponding author (zl249@ibcas.ac.cn).

## Abbreviations

CCD: Carotenoid cleavage dioxygenase
CRISPR/Cas9: clustered regulatory interspaced short palindromic repeats/CRISPR-associated protein 9
EGFP: Enhanced green fluorescent protein
GM: Glycerol-maltose medium
hptII: hygromycin phosphotransferase II
MAX: More axillary branching
NAA: Naphthalene acetic acid
NOA: Naphthoxy acetic acid
qRT-PCR: Quantitative real-time PCR
sgRNA: Single guide RNA
SLs: Strigolactones
