# Supplementary material for: Knockout of VvCCD8 gene in grapevine affects shoot branching
Source: BMC Plant Biol. 2020 Jan 29;20:47. doi: 10.1186/s12870-020-2263-3 (PMC6990564; doi:10.1186/s12870-020-2263-3)
Supplement: Supplementary file 1 — Additional file 1: Figure S1. Sequencing results of VvCCD7 and VvCCD8 fragments amplified from 41B cells. a The target sequence of VvCCD7 gene in 41B. b The target sequence of VvCCD8 gene in 41B. The target sites in VvCCD7 and VvCCD8 genes are highlighted in dark blue. [file 12870_2020_2263_MOESM1_ESM.docx]

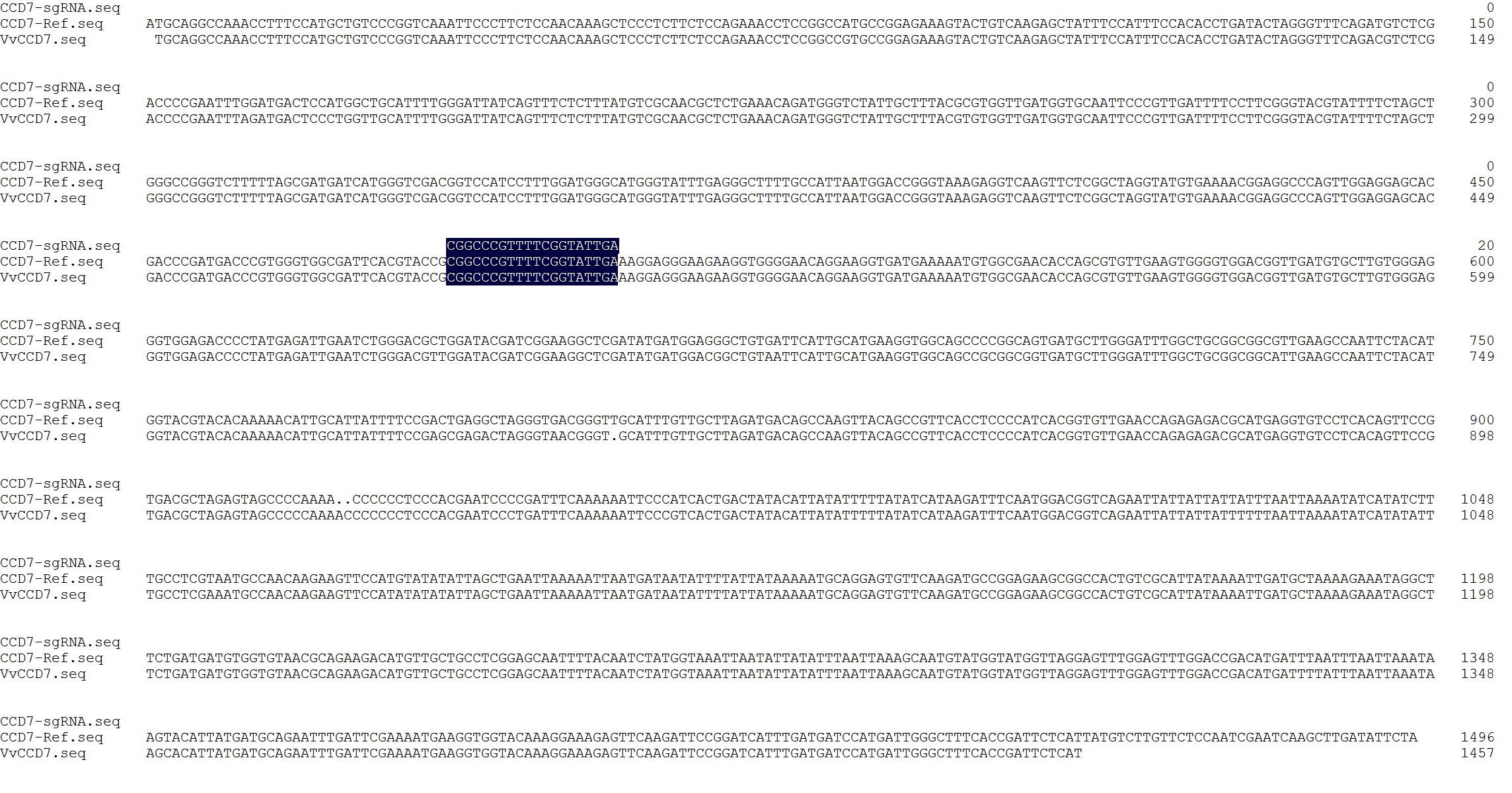


**a**


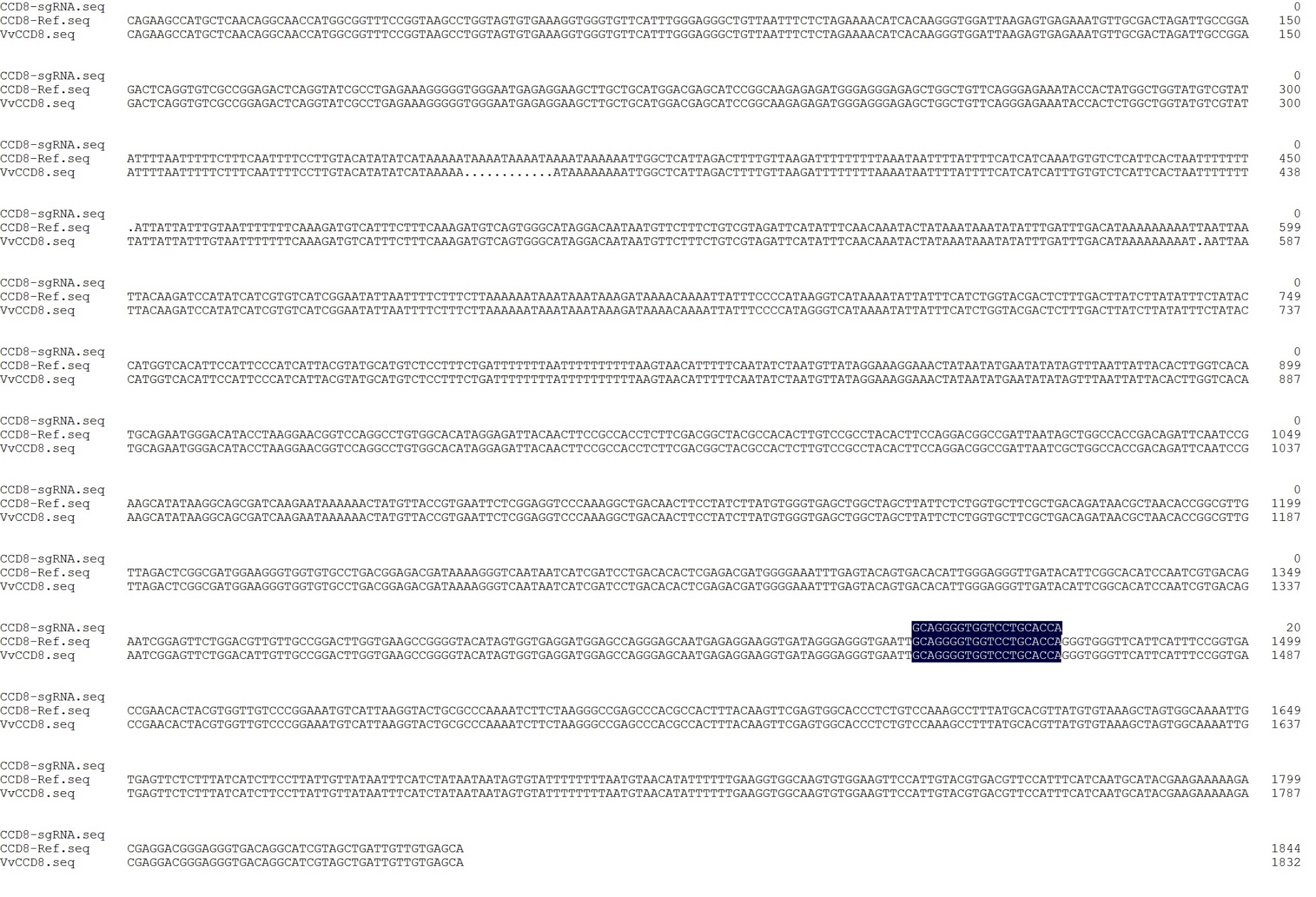


**b**

**Figure S1** Sequencing results of *VvCCD7* and *VvCCD8* gene fragments amplified from 41B cells. **a** The target sequence of *VvCCD7* gene in 41B. **b** The target sequence of *VvCCD8* gene in 41B. The target sites in *VvCCD7* and *VvCCD8* genes are highlighted in dark blue
