# Supplementary material for: Knockout of VvCCD8 gene in grapevine affects shoot branching
Source: BMC Plant Biol. 2020 Jan 29;20:47. doi: 10.1186/s12870-020-2263-3 (PMC6990564; doi:10.1186/s12870-020-2263-3)
Supplement: Supplementary file 2 — Additional file 2: Figure S2. Sequencing results of the two putative off-target sites in VvCCD8 knockout lines. The two off-target sites predicted within exons of other genes were selected for off-target analysis. Two VvCCD8 knockout lines, Plant #1 and Plant #6 were used in the experiment. The amplified fragments containing the off-target sites were amplified and cloned into pLB-Simple vector. At least 6 clones for each site were used for Sanger sequencing. [file 12870_2020_2263_MOESM2_ESM.docx]

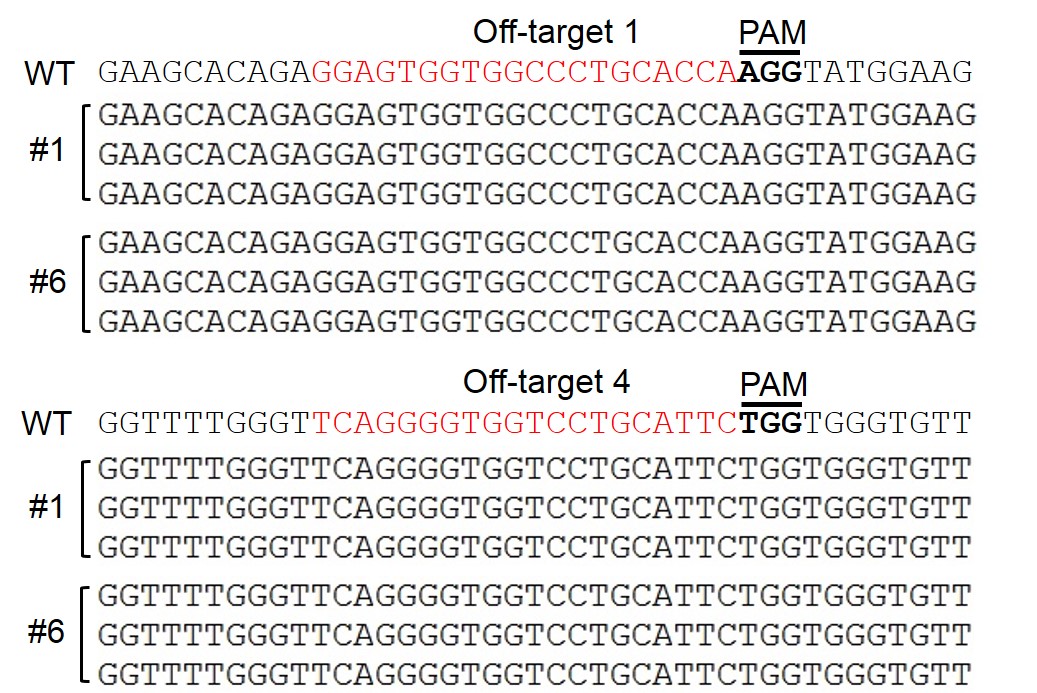


**Figure S2** The sequencing results of two potential off-target sites in *VvCCD8* knockout lines. The two off-target sites predicted within exons of other genes were selected for off-target analysis. Two *VvCCD8* knockout lines, Plant #1 and Plant #6 were used in the experiment. The amplified fragments containing the off-target sites were amplified and cloned into pLB-Simple vector. At least 6 clones for each site were used for Sanger sequencing
