# Supplementary material for: Knockout of VvCCD8 gene in grapevine affects shoot branching
Source: BMC Plant Biol. 2020 Jan 29;20:47. doi: 10.1186/s12870-020-2263-3 (PMC6990564; doi:10.1186/s12870-020-2263-3)
Supplement: Supplementary file 3 — Additional file 3: Figure S3. The original gel image of PCR identification of T-DNA insertions in CCD8-sgRNA plants. The vector plasmid (P1) and the transgenic cells (P2) were used as the positive controls, while wild-type plant was used as the negative control (N). Lanes 1–6 represent individual CCD8-sgRNA plants. [file 12870_2020_2263_MOESM3_ESM.docx]

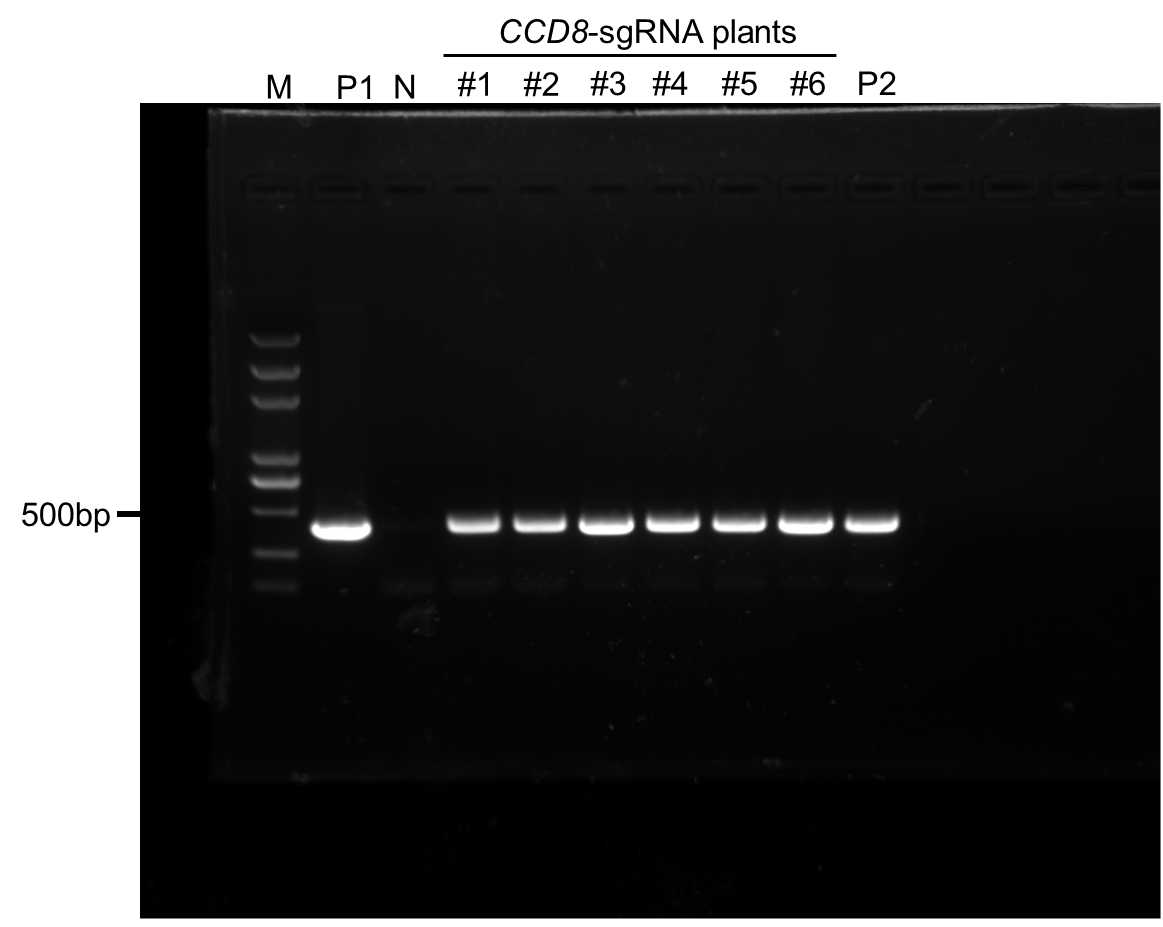


**Figure S3** The original gel image of PCR identification of T-DNA insertions in *CCD8*-sgRNA plants. The vector plasmid (P1) and the transgenic cells (P2) were used as the positive controls, while wild-type plant was used as the negative control (N). Lanes 1-6 represent individual *CCD8*-sgRNA plants
