# Supplementary material for: Knockout of VvCCD8 gene in grapevine affects shoot branching
Source: BMC Plant Biol. 2020 Jan 29;20:47. doi: 10.1186/s12870-020-2263-3 (PMC6990564; doi:10.1186/s12870-020-2263-3)
Supplement: Supplementary file 4 — Additional file 4: Table S1. List of primers used in this study. [file 12870_2020_2263_MOESM4_ESM.docx]

Table S1 List of primers used in this study

| Primer name | Sequence (5’-3’) | Experiment |
| --- | --- | --- |
| CCD7-F | TGCAGGCCAAACCTTTCCAT | PCR Cloning |
| CCD7-R | ATGAGAATCGGTGAAAGCCCA |  |
| CCD8-F | CAGAAGCCATGCTCAACAGG |  |
| CCD8-R | TGCTCACAACAATCAGCTACG |  |
| EGFP-F | CACCAGTCTCTCTCTATGGTGAGCAAGGGCGAGGA |  |
| EGFP-R | TTATTATGGAGAAACTTACTTGTACAGCTCGTCCA |  |
| CCD7-sgRNA-R | GCTATTTCTAGCTCTAAAACCGGCCCGTTTTCGGTATTGACAATCACTACTTCGACTCT | Vector Construction |
| CCD8-sgRNA-R | GCTATTTCTAGCTCTAAAACTGGTGCAGGACCACCCCTGCAATCACTACTTCGACTCT |  |
| Cas9-PCR-F | ACCTTCCGCATCCCCTACTA | Identification of exogenous T-DNA |
| Cas9-PCR-R | ATTTCCACGGAGTCGAAGCA |  |
| Actin1-qPCR-F | CAGCAGATGTGGATCTCAAA | Quantitative real-time PCR |
| Actin1-qPCR-R | CTGTGGACAATGGAAGGAC |  |
| CCD8-qPCR-F | TATGCCTATGCTTGTGGGGC |  |
| CCD8-qPCR-R | CGAGGGCACAGAACCTTCAT |  |
| CCD8-OFF1-F | GGGCAGTGACAAAAACCGTG | Detection of putative off-target sites |
| CCD8-OFF1-R | TTCGACTCGGACATCACCTG |  |
| CCD8-OFF2-F | CGCAGATCACGTAATCAAGCTC |  |
| CCD8-OFF2-R | ATGAGAAAGAGAAAGACCAGTTGTA |  |
