# Supplementary material for: Knockout of VvCCD8 gene in grapevine affects shoot branching
Source: BMC Plant Biol. 2020 Jan 29;20:47. doi: 10.1186/s12870-020-2263-3 (PMC6990564; doi:10.1186/s12870-020-2263-3)
Supplement: Supplementary file 5 — Additional file 5: Table S2. Putative off-target sites predicted for CCD8-sgRNA. [file 12870_2020_2263_MOESM5_ESM.docx]

Table S2 Putative off-target sites predicted for *CCD8*-sgRNA

| Off-target site | Sequence | Locus | Gene | Region |
| --- | --- | --- | --- | --- |
| 1 | GGAGTGGTGGCCCTGCACCAAGG | 3:-7303784 | VIT_03s0091g00830 | Exon |
| 2 | ACATGTGTGGTCCTGCACAAGGG | 14:-25629282 | - | Intergenic |
| 3 | TCAGGGGTGGTCCTGCATTCTGG | 13:-20544201 | - | Intergenic |
| 4 | TCAGGGGTGGTCCTGCATTCTGG | 13_random:+2829395 | VIT_13s0019g01150 | Exon |
| 5 | ACAGGGTTGGTCCTGCCCCTTGG | Un:+20214144 | - | Intergenic |
